# Fingerprint of semi-crystalline structure memory in the thermal and ionic conduction properties of amorphous ureasil–polyether hybrid solid electrolytes†

**DOI:** 10.1039/d1ra09138g

**Published:** 2022-02-14

**Authors:** Gustavo Palácio, Sandra H. Pulcinelli, Celso V. Santilli

**Affiliations:** Chemistry Institute of the São Paulo State University, UNESP 14800-060 Araraquara São Paulo Brazil cv.santilli@unesp.br

## Abstract

Correlations among the structure, thermal properties, and ionic conductivity of solid polymer electrolytes (SPEs) were studied using a ureasil–polyethylene oxide (U-PEO) organic–inorganic hybrid prepared according to a simple sol–gel route, employing a low molecular weight PEO macromer (*M*_w_ = 1900 g mol^−1^). The behavior of an amorphous sample loaded with lithium triflate (LiTFSI) at an optimum ratio between ether oxygen and lithium (EO/Li^+^ = 15) was compared with that of a semicrystalline sample prepared without salt loading. The temperature range investigated by differential scanning calorimetry (DSC), Raman spectroscopy, small angle X-ray scattering (SAXS), and complex impedance spectroscopy covered both the glass transition and the melting temperature of the U-PEO. The gauche to trans conformational transformation of the (O–C–C–O)Li^+^ sequence showed similarity between the temperature evolution of the semi-crystalline U-PEO and amorphous U-PEO:Li^+^ samples, providing an indication of the local structural memory of crystalline state in the amorphous SPE. The linear thermal expansion of the average correlation distance between the siloxane crosslink nodes and the long-distance period of the lamellar semi-crystalline edifice were determined by SAXS. Comparison of the expansion curves suggested that although the siloxane nodes were excluded from the PEO crystalline edifice, the sharp expansion of the amorphous region between the lamellae during melting permitted modulation of the free volume of the hybrid network. In addition, the temperature-induced Li^+^-EO decomplexation observed by Raman spectroscopy explained the change of the average activation energy of the conduction process revealed by the different Arrhenius regimes. These results evidence the key role of the ionic conductivity decoupling from the segmental motion of chain pair channels on the improvement of ion mobility through the free volume between chains. This concept may inspire materials chemistry researchers to design optimized structures of polymer electrolytes with minimized structural memory of crystaline building blocks and improved ionic conductivity.

## Abstract 2

Ionic conduction of amorphous ureasil–polyelectrolytes containing Li^+^ cations (U-PEO:LiTFSI) affected by the persistent PEO chain building blocks of the crystalline polymer.

## Graphical abstract

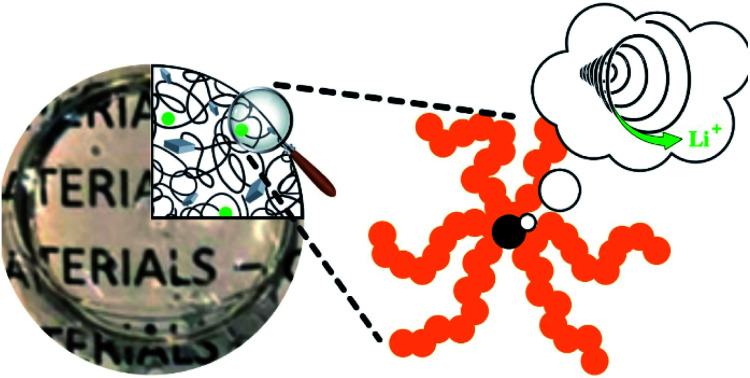

## 1. Introduction

The research and development concerning solid-state electrolytes (SEs) for electrochemical devices such as lithium-ion batteries (LIBs) has remained popular, due to their better performance in terms of safety and fabrication, compared to liquid electrolytes that can have associated safety concerns due to possible leakage and the explosive nature of volatile organic electrolytes.^1,2^ Most liquid electrolytes used in commercial LIBs are non-aqueous solutions, typically containing a lithium salt such as lithium triflate (LiTFSI), due to the “soft” characteristic of the CF_3_SO_3_^−^ anion, which has low ion-dipole stabilization energy and high solvation energy, due to its mutual polarizability, tending to form free ions.^3^ The development of solid-state polymer electrolytes (SPEs) has evolved rapidly,^1,3–6^ after the first studies by Wright^7^ in 1973 concerning the ionic conductivity of lithium salt dissolved in poly(ethylene oxide) (PEO). Renewed interest in novel SPEs has been stimulated by the development of lithium metal batteries (LMBs) of high specific capacity, due to the increasing demand for electric vehicles, portable electronic equipment, and implantable medical devices.^8,9^ In PEO, the high segmental motion of the chains at temperatures above the glass transition results in high ionic mobility, but harms the mechanical properties of SPEs.^8^ In addition to the concurrence between these properties, PEO is semi-crystalline and the Li^+^ transport is limited to the mobility of its amorphous phase. Hence, unmodified PEO must be heated to around 80 °C to achieve conductivity higher than 10^−4^ S cm^−1^, which is a limitation for many applications.^10^ Nonetheless, despite the limitations of PEO, materials based on modified PEO are still considered the best SPE candidates, because they are inexpensive and nontoxic. Various strategies can be used to modify the PEO architecture (comb copolymers and block copolymers with micro domain structures), among which organic–inorganic hybrid (OIH) materials are of particular interest.^11^ These materials provide high capacity for the solubilization of Li^+^ salts, dimensional stability resulting from the network formed by the hard inorganic cross-linkers, and the possibility of controlling the coordination of counter-ions to the ligand present at the soft–hard interface.^12^

The sol–gel process is well suited to the production of rubber OIH materials presenting ion-conducting properties and liquid-like diffusive behavior.^13^ The OIH material studied here, U-PEO, prepared the first time by Hall *et al.*^14^ and Smid *et al.*,^15^ followed by Spindler and Shriver,^16^ consists of a rubber network composed by short PEO chains (1900 g mol^−1^) covalently bonded to siloxane nodes by urea groups forming ureasil cross-linkers (U).^17^ The expected synergistic contribution of each part of the framework causes complex structural effects arising from interaction of the alkaline salt with the PEO chains and the urea bridge structure.^18^ The loading of U-PEO with a lithium salt has several effects. Firstly, weakening or breaking of the polyether–urea hydrogen bonds occurs due to the complexation involving anions with Lewis acid character,^12^ increasing the lithium transference number.^8,19,20^ Secondly, strong solvation of Li^+^ by the ether type oxygen reduces the amount of macroscopic crystalline phase to zero, which eliminates the anisotropy effect of transport through the amorphous phase confined in the randomly orientated crystalline lamellae. This contributes to improving the cation motion, assisted by segmental motion at moderate Li^+^ concentrations with [EO]/[Li^+^] > 15.^21–23^ On the other hand, for [EO]/[Li^+^] < 15, the conductivity decreases, due to the formation of O–Li^+^–O cross-linking between ether-type oxygen atoms of different chains. Accordingly, maximum ionic conductivity occurs for the ratio [EO]/[Li^+^] = 15.^23^

**Fig. 1:**
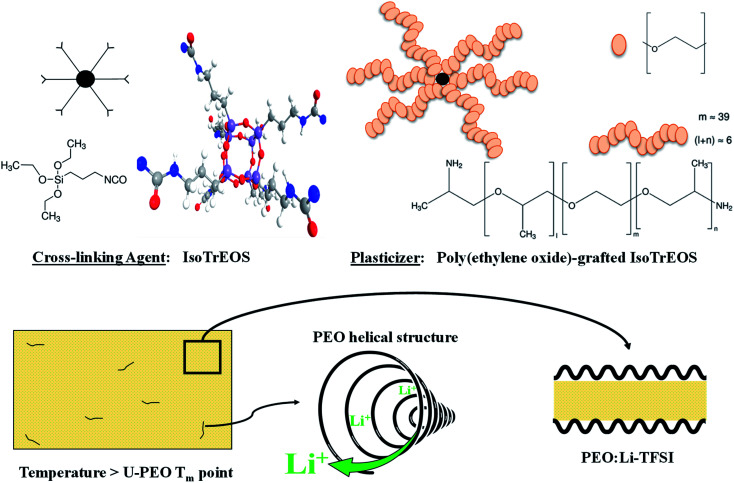
Scheme of the PEO mesostructural organization and tubular cylindrical geometry of the (PEO)_6_:LiTFSI complex above the PEO melting temperature.

In summary, the electrical properties of U-PEO are strongly dependent on the mobility of both the polymer chains and the active ionic species, which themselves depend on the polymer mesostructural organization resulting from the complexation induced by the lithium salt. In the case of PEO/LiTFSI with PEO of low molecular weight (*M*_w_), a crystallization gap was observed for [EO]/[Li^+^], ranging from 12 : 1 to 6 : 1.^24–26^ However, it was suggested that crystalline structures of PEO lamellae and tubular cylindrical geometry of the (PEO)_6_:LiTFSI complex persisted in the fully amorphous material, due to a memorizing capacity of polymers, when low molecular weight resulted in the structural elements shown schematically in Fig. 1.

Reiter^27^ has defined the term ‘memory’ as the ability of the material to establish a link between past and future in terms of its properties. Nevertheless, it is possible to erase the all memory information of a polymeric material by employing an appropriate kinetics of annealing and relaxation.^27^ However, the time required to reach a thermal equilibrated state, ensuring the erasure of information, may be exceedingly long.^28^ As a result, polymeric materials can never reach this thermal equilibrated point and, remembering their thermal and mechanical history, as example, the structure and conformation in which they were synthesized.

Computational simulations^29,30^ and analytical statistics of mechanical theories^31–33^ have clarified the major factors for the memory of conformation in polymers.^34^ One of the factors was the interaction between the monomers with different conformational energies, which are able to memorize their conformation in a condensed state. The difference of the energies is due to the heterogeneous interactions created by different conformations, being some of them potentially more favorable than others.^35^ Flexible polymers like PEO and PPO poly(propylene oxide) have a strong coupling of ionic transport to structural relaxation and good ion solvation.^36^ For example the (PEO)_4_:LiClO_4_ shows a decoupling of the structural and conductivity relaxations in a temperature range below the PEO melting point, due to the semi-crystalline nature of PEO, which hinders long-range ion-migration below its melting temperature. Even after considerable efforts of the scientific community, Gao, *et al.* have recently published a review discussing the perspectives on current challenges and future directions of SPEs.^37^ In their conclusions, the ionic conductivity at room temperature and below is still unsatisfactory for their practical applications.

The goal of this paper is to establish new links between the molecular and mesoscopic structures and the macroscopic properties. To reach this objective, we investigated the correlation among the structural, thermal, and ion conduction properties, by means of a comparative study of the temperature evolution of semi-crystalline U-PEO (PEO *M*_w_ = 1900 g mol^−1^) and an SPE based on U-PEO:LiTFSI with [EO]/[Li^+^] = 15. This approach provided evidences of the existence of semi-crystalline structural elements memory, which influenced the macroscopic properties of the amorphous SPE. We showed through the experimental results that the decrease of Li^+^-EO complexation on the channels formed by the PEO chain pairs induce additional improvement on the ionic conductivity.

## 2. Experimental

### 2.1. Sample preparation

The U-PEO materials were synthesized by the sol–gel method, using a well-known procedure^38,39^ and commercially available reagents. In brief, the ureasil (U) cross-linking agent (3-(isocyanatopropyl)-triethoxysilane (IsoTrEOS), Fluka, Aldrich, 95% purity, CAS #24801-88-5) was covalently bonded to both ends of the functionalized PEO (O,O′-bis(2-aminopropyl) polypropylene glycol-*block*-polyethylene glycol-*block*-polypropylene glycol, *M*_w_ = 1900 g mol^−1^, Jeffamine® ED-2003, CAS #65605-36-9) by reacting their terminal aminopropyl groups, in a molar ratio of 1 : 2. These reagents were stirred together in ethanolic solution, under reflux for 6 h at 78 °C, to prepare 20 g of the OIH precursor (EtO)_3_Si-(CH_2_)_3_NHC(=O)NHCH(CH_3_)-(polyether)-OCH_2_CH(CH_3_)NH(O=)NHC(CH_2_)_3_-Si(OEt)_3_. The second step comprised a sol–gel reaction involving Si(OEt)_3_ hydrolysis, generating silanol moieties, followed by a condensation reaction to form ureasil cross-linking nodes. The hydrolysis was started by adding 30 μL of water, to satisfy the ratio [H_2_O]/[Si] = 4, and 12 μL of HCl (10^−2^ mol L^−1^) as catalyst, to 1.6 mL of the ethanolic solution containing the hybrid precursor (equivalent to 0.5 g of the solid precursor). In the case of the Li^+^-loaded sample (U-PEO:Li^+^), addition was also made of 0.176 g of CF_3_SO_2_NLiSO_2_CF_3_ (Fluka, Aldrich, 99.95% purity, CAS #90076-65-6). The lithium content was calculated considering the ratio [EO]/[Li^+^] = 15. Transparent rubber xerogels with mass of around 0.5 g were obtained after drying at room temperature for 48 h.

### 2.2. Characterization

All the measurements were performed as a function of temperature, starting with an isothermal period of 10 min at +100 °C, in order to eliminate adsorbed water molecules, followed by fast cooling to ≈−100 °C, and controlled heating to +100 °C. The thermal, vibrational, nanostructural, and electrical properties were determined as described below.

Differential scanning calorimetry (DSC) measurements were carried out using a Q100 analyser (TA Instruments). Small disks with diameter of 0.5 cm and weighting ≈10 mg were cut from the OIH samples and placed in aluminium pans. The analyses were performed under nitrogen flow of 50 mL min^−1^.

Raman spectra were recorded in backscattering geometry, using a Raman RXN1 analyser (Kaiser Optical Systems, Inc.). The instrument incorporated a near-infrared laser diode, operating at 785 nm, and a HoloPlex transmission grating that diffracted the different wavelengths of the polychromatic Raman scattered light into different angular output paths on the Peltier-cooled charge coupled device detector. The measurements were performed in the spectral range from 200 to 1000 cm^−1^, at a resolution of 2 cm^−1^. Raman spectra were recorded every 12 s during heating at a programmed rate, resulting in collection of one spectrum for each degree (°C).

The nanostructures of the hybrids were analysed by small angle X-ray scattering (SAXS) measurements, performed at the SAXS1 beamline of the National Synchrotron Light Laboratory (LNLS, Campinas, Brazil). The beamline was equipped with a 2D Pilatus 300k detector located 910.9 mm from the sample, recording the image of the scattering intensity, *I*(*q*), as a function of the modulus of the scattering vector, *q* = (4π/*λ*)sin(*ε*/2), where *ε* is the X-ray scattering angle. The data were normalized considering the varying intensity of the direct X-ray beam, detector sensitivity, and sample transmission. The intensity of parasitic scattering due to the cell windows and vacuum was subtracted from the total scattering intensity. Small pieces of the monolithic hybrids were introduced into a temperature-controlled sample holder (model DSC6000, Linkan) allowing control of the temperature in the range from −100 °C to 100 °C. SAXS patterns were recorded every 12 s during heating, taking one measurement every degree (°C).

The ionic conductivity of the U-PEO:Li^+^ SPE was measured using a vacuum system, with an impedance/gain phase analyser (model SI1260, Solartron) connected to a programmable temperature controller (model K-20, MMR Technologies, Inc.). The measurements were made in the frequency range from 1 MHz to 50 Hz, with applied signal amplitude of 3 mV. The sample was prepared in the form of a rectangular monolith about 2 mm thick, with flat surfaces and electrical contact area of 3 mm^2^. The two parallel surfaces of the sample were painted with a silver ink (type #09937, Sigma-Aldrich). To form the electrical contacts, two copper wires were connected to the electrical active area using Leit-C adhesive (type #09929, Sigma-Aldrich), with each end being welded to the electrical terminals of the sample holder. The part of the sample without the electrical contact was glued to the heating source of the sample holder using a silicone heat sink, in order to assist energy transfer. The Nyquist plots (*Z*′ *versus Z*′′), obtained every 10 °C, were normalized by the electrolyte geometric factor of the solid monoliths. Z-view software^40^ was used to analyse the Nyquist plots obtained from the impedance measurements.

## 3. Results & discussion

### 3.1. Effect of Li^+^ loading on U-PEO thermal and structural transformations

The U-PEO and U-PEO:Li^+^ DSC curves (Fig. SI.1†) displayed a common characteristic, which was the relatively sharp discontinuity due to the heat capacity variation (Δ*C*_p_), characteristic of a second order transition, ascribed to the glass transition (*T*_g_).^3^ The *T*_g_ event occurred at −58 and −41 °C for U-PEO and U-PEO:Li^+^, respectively. The upshift of the *T*_g_ value evidenced increased rigidity of the polymer chains, due to the formation of inter- and/or intramolecular interactions between the lithium cations and the ether-type oxygen atoms (Li-OEt) of the amorphous phase of the PEO macromer.^12^ The U-PEO endothermic event typical of a first order transition occurred between 0 and 35 °C, with a maximum at ∼29 °C, corresponding to the melting temperature (*T*_m_) of the semi-crystalline PEO.^41^ After loading with Li^+^, the melting event was absent, indicating that the Li^+^ cations hindered PEO crystallization, due to the Li-OEt coordination that induced crown-ether conformations, restricting the helical PEO conformations of the crystalline phase.^41^

**Fig. 2:**
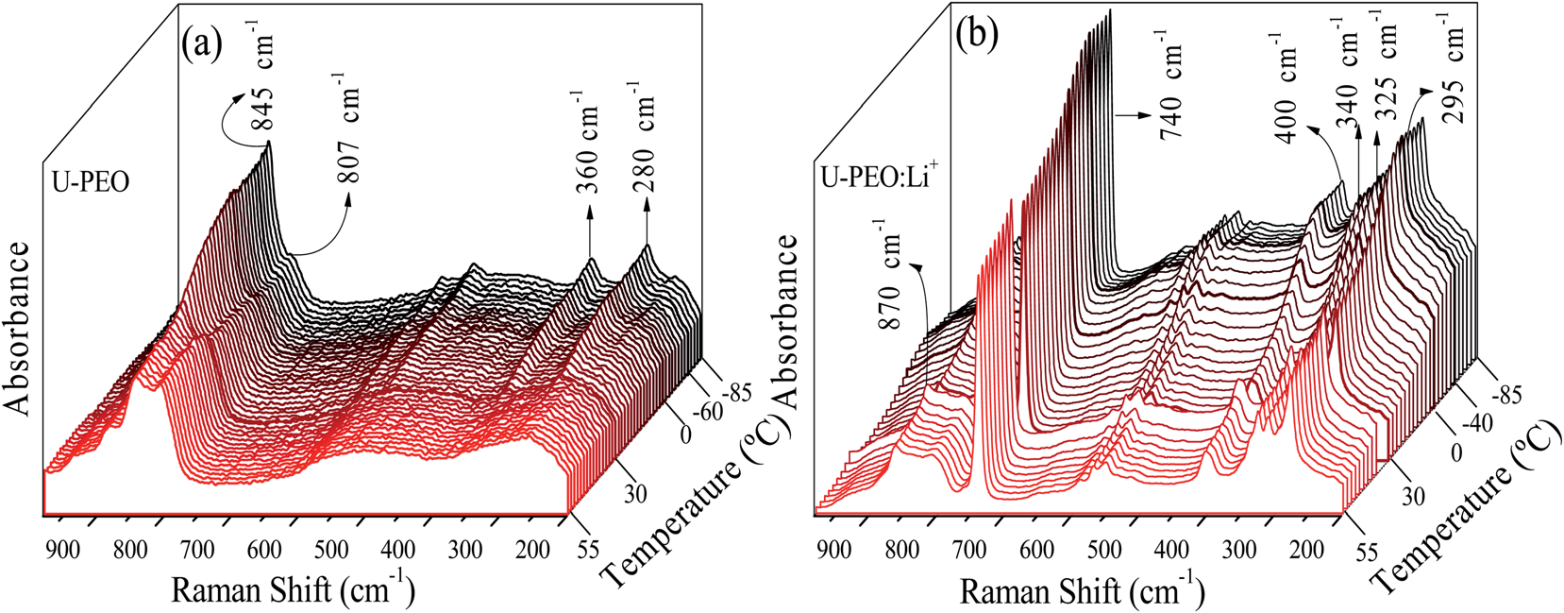
Stacked Raman spectra for (a) U-PEO and (b) U-PEO:Li^+^, recorded *in situ* during heating from −85 to 55 °C.

In order to analyse the effect of Li^+^ loading on conformational changes of the PEO chains of the U-PEO host during the melting and glass transitions, Raman spectra were acquired as a function of temperature from −85 to 55 °C. In the low temperature range (*T* ≤ 10 °C, Fig. 2(a)) the U-PEO spectra showed an intense band at around 845 cm^−1^, attributed to the gauche conformation of the PEO backbone (O–C–C–O). The contribution of this band decreased continually in the temperature range of the melting event revealed by DSC, accompanied by a small increase of the band at around 807 cm^−1^, for *T* > 29 °C, attributed to the trans backbone conformation.^42^ In the same temperature range as the melting event (Fig. SI.1†), the two medium intensity bands at 280 and 360 cm^−1^ disappeared. These two contributions, corresponding to CCO and COC bending vibrations,^43^ confirmed the *gauche* → *trans* conformation shift of the PEO backbone during the solid → liquid transition.

For U-PEO:Li^+^, the backbone (O–C–C–O) conformation Raman bands remained almost invariant in the entire temperature range studied (Fig. 2(b)), confirming the absence of PEO crystalline phase evolution. An intense peak at around 740 cm^−1^ could be ascribed to symmetric stretching of SNS from the triflate anion,^44^ confirming dissociation of the salt in the OIH host matrix. In the region dominated by the spectral pattern of the triflate anion, between 260 and 410 cm^−1^, bands at 295, 325, and 340 cm^−1^ could be ascribed to SO_2_ rocking modes,^43^ while a band at around 275 cm^−1^ could be attributed to the CF_3_ rocking mode.^45^ For U-PEO:Li^+^, the absence of the bands at 280 and 360 cm^−1^, related to gauche CCO and COC bending vibrations, suggested dominance of the *trans* (O–C–C–O) backbone conformation for this hybrid electrolyte, even below the melting temperature. This feature evidenced the amorphization induced by Li^+^ loading in the U-PEO matrix, in agreement with the DSC results.

**Fig. 3:**
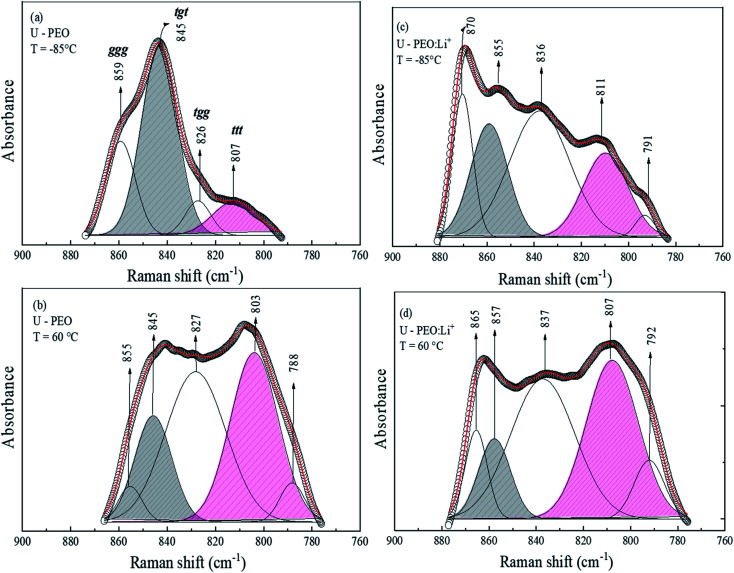
Gaussian fitting of the Raman band envelope in the region of the CH_2_ rocking vibration, recorded at a temperature below the glass transition and above the melting point for U-PEO at (a) *T* = −85 °C and (b) *T* = 60 °C, and for U-PEO:Li^+^ at (c) *T* = −85 °C and (d) *T* = 60 °C.

As evidenced in Fig. SI.1† and in agreement with the literature,^41^ the addition of an ionic salt containing a small cation promoted crown-ether type coordination (*trans* conformations). Crown-ether conformations deviate from the helical PEO conformations typically found in bulk PEO crystals.^46–48^ For in-depth investigation of the effective conformation of the (O–C–C–O) sequence of PEO polymers containing small cations, the individual components in the region of the CH_2_ rocking vibration (770–900 cm^−1^) were calculated, as a function of temperature. The nonlinear least-square curve fitting was based on the number and positions of the individual bands found by the well-established criterion of the second derivative of the spectrum.^49^ The results, summarized in Fig. 3, revealed that the conformation window of the U-PEO spectrum presented four components at low temperature and five components at high temperature, assigned to various conformational modes (*ttt*, *tgt*, *tgg*, and *ggg* triads of *trans* (*t*) and *gauche* (*g*) C–C bands).^48^ However, the most important observed effects were the intensity changes with increase of the temperature, for the components at 807, 845, and 880 cm^−1^, ascribed to *trans* and *gauche* conformations of the (O–C–C–O) backbone, respectively. Fig. 3(c) shows the dominant contribution of a band at 870 cm^−1^, assigned to M–O_*n*_ breathing motion in the PEO:Li^+^ complexes,^50^ while absorbance of bands corresponding to *trans* (807 cm^−1^) and *gauche* (855 and 837 cm^−1^) conformations were comparable. The U-PEO:Li^+^ Raman spectra at high temperature (Fig. 3(d)) also revealed a changing profile, suggesting an increase of the *trans* conformation contribution at the expense of decomplexation and *gauche* state abundance, as evidenced by the similar decrease of the bands at 865 and 857 cm^−1^.

**Fig. 4:**
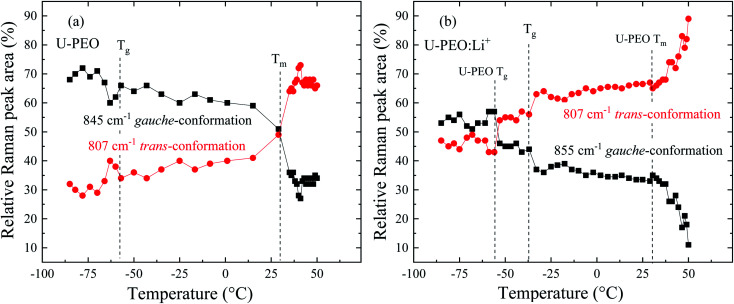
Evolution of the relative areas (%) of Raman bands corresponding to *gauche* (845 cm^−1^) and *trans* (807 cm^−1^) conformations of the (O–C–C–O) backbone, as a function of temperature, for (a) U-PEO and (b) U-PEO:Li^+^.

In order to rationalize the analysis of this complex behavior, the relative areas (%) of the Raman bands for the *trans* and *gauche* components were investigated in the temperature range corresponding to the glass transition and melting, as shown in Fig. 4(a) and (b) for U-PEO and U-PEO:Li^+^, respectively. Fig. 4(a) shows almost invariance of the areas of the bands at 845 and 807 cm^−1^ at lower temperature, with an abrupt change near the *T*_g_ event. Above *T*_g_, a small decrease of the *tgt* band at 845 cm^−1^ occurred concomitantly with increase of the *ttt* band at 807 cm^−1^. This almost linear evolution accelerated near *T*_m_, leading to a crossover point at 15 °C, the beginning of PEO melting. A plateau occurred above 35 °C, in the temperature range where the sample was completely amorphous. The sharp changes observed in the same region as the melting event could be ascribed to the mostly *gauche* → mostly *trans* conformational transition. It is noteworthy that the overall temperature evolution of the both conformation curves display bottleneck shapes near to the *T*_g_ and *T*_m_, similar to the entropy-energy funnel theoretically proposed for folding–defolding of helicoidal segments of protein.^33^

Fig. 4(b) shows quite different profiles, but with comparable temperature evolutions. The areas of both bands of U-PEO:Li^+^ were essentially invariant below −58 °C, compared to the unloaded U-PEO, with a sharp inversion of intensity near the U-PEO *T*_g_ (−58 °C), suggestive of a PEO backbone conformational memory effect. This phenomenon can be explained by the chain connectivity interplay and the excluding volume constraints, with leads to formation of an energetically favorable conformation of pairs of mere, hindering the formation of other such pairs.^35^

A second abrupt change occurred near the *T*_g_ of U-PEO:Li^+^ (−41 °C), with decreases of the bands at 855 and 870 cm^−1^, occurring concomitantly with increase of the *ttt* band at 807 cm^−1^, which was similar to the ‘bottlenecks’ evolution observed for U-PEO between *T*_g_ and *T*_m_ (Fig. 4(a)). However, above the host U-PEO melting point, accelerated changes of the band areas were observed for U-PEO:Li^+^. These three accelerating events suggested the existence of a memory effect of the Li^+^-free polymeric chains in the (O–C–C–O) backbone conformation of the U-PEO:Li^+^ sample. This finding indicated that the extent of the amorphization/disorganization induced by Li^+^ loading also depended on the cation–decomplexation equilibrium kinetic, which appeared to be more effective above the *T*_m_ point of the unloaded U-PEO hybrid. This is a typical manifestation of the entropic contribution to the kinetically organized elements of the crystalline structure.^33,35^

### 3.2. Effect of Li^+^ loading and temperature on the U-PEO nanostructure

**Fig. 5:**
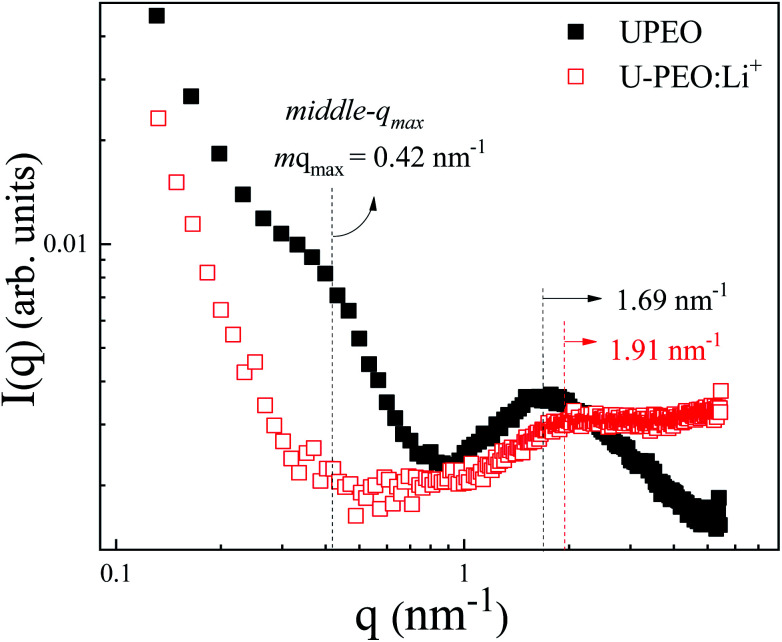
Room temperature SAXS curves, using a log–log scale, for the U-PEO (full black squares) and U-PEO:Li^+^ (empty red squares) samples.

SAXS measurements were carried out to investigate the effects of Li^+^ loading and phase transformation on the nanostructural features of the OIH. The room temperature SAXS pattern for U-PEO (Fig. 5) presented a broad peak ascribed to the interference effect of X-ray scattering by the spatially correlated siloxane crosslink nodes.^51^ The average correlation distance between two adjacent siloxane nodes (*d*_sil_) could be calculated from the position of the peak maximum (*q*_max_): *d*_sil_ = (2π)/*q*_max_.^52^ After Li^+^ loading, decreases of *d*_sil_ (from 3.72 to 3.29 nm) and peak intensity (*I*(*q*_max_)) were observed. This behavior was associated with decrease of the electron density contrast between the siloxane nodes and the polyether matrix, due to the interaction of Li^+^ with the ether oxygen of the PEO (Li-OEt), decreasing the free volume between the oligomeric chains.^3^

The U-PEO SAXS pattern revealed a poorly defined second maximum located in the middle *q* range (*q* < 1 nm^−1^, with *q*_max_ ∼0.42 nm^−1^), which was not observed for the Li^+^-loaded sample. This maximum corresponded to a Bragg *d*_sc_ spacing of 14.9 nm, which was similar to the long-period distance of superstructures formed by lamellar arrangements of crystalline and amorphous parts of semi-crystalline PEO homopolymers.^53,54^ The origin of this maximum was confirmed from the temperature dependency of the SAXS curves for the U-PEO and U-PEO:Li^+^ samples.

**Fig. 6:**
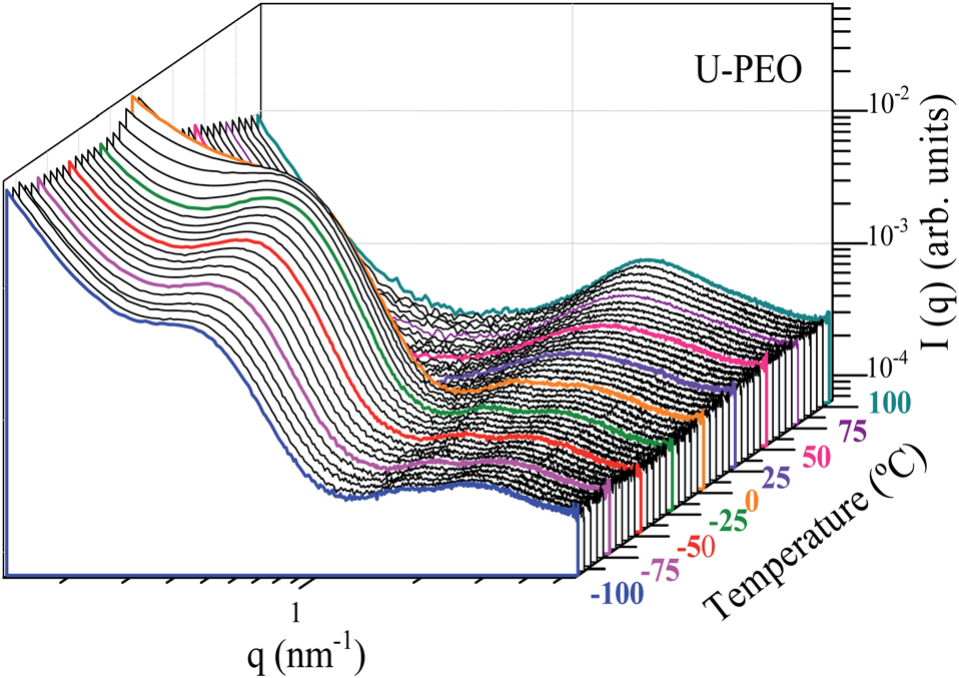
Stacked log–log SAXS curves for U-PEO, as a function of temperature, recorded *in situ* during heating from −100 to 100 °C.

Fig. 6 and SI.2† show the evolution, according to temperature, of the SAXS curves recorded *in situ* during heating of the U-PEO and U-PEO:Li^+^ samples, respectively. In the temperature range −100 °C < *T* < ∼29 °C, the SAXS curves exhibited the two maxima discussed earlier, with one in the middle *q* range (*q* < 1 nm^−1^) and the other in the high *q* range (*q* > 1 nm^−1^). The former vanished above the PEO melting point (*T*_m_ = 15 °C), confirming the attribution of this peak to the long-distance period of the semi-crystalline PEO. In addition, this feature was in full agreement with the temperature dependence of the PEO organic chain conformation, revealed from the Raman spectra (Fig. 2(a) and 4(a)), which showed *gauche* → *trans* state transition. It could be concluded from this that the long-distance period peak, found for the U-PEO hybrid host, had a backbone dependence similar to that reported for the PEO homopolymer.^55^ In contrast, in this temperature range, no remarkable modification was observed in the SAXS curves for U-PEO:Li^+^, confirming the amorphous nature of the Li^+^-loaded hybrid (Fig. SI.3†). However, both the unloaded and Li^+^-loaded hybrids showed some correlation peak features in common, such as downshift of *q*_max_ and increased intensity as the temperature increased. These features were associated with the thermal expansion of the polymeric framework, which led to increase of the electron density contrast between the PEO and the siloxane nodes, due to the greater contribution of the free volume between the chains.

**Fig. 7:**
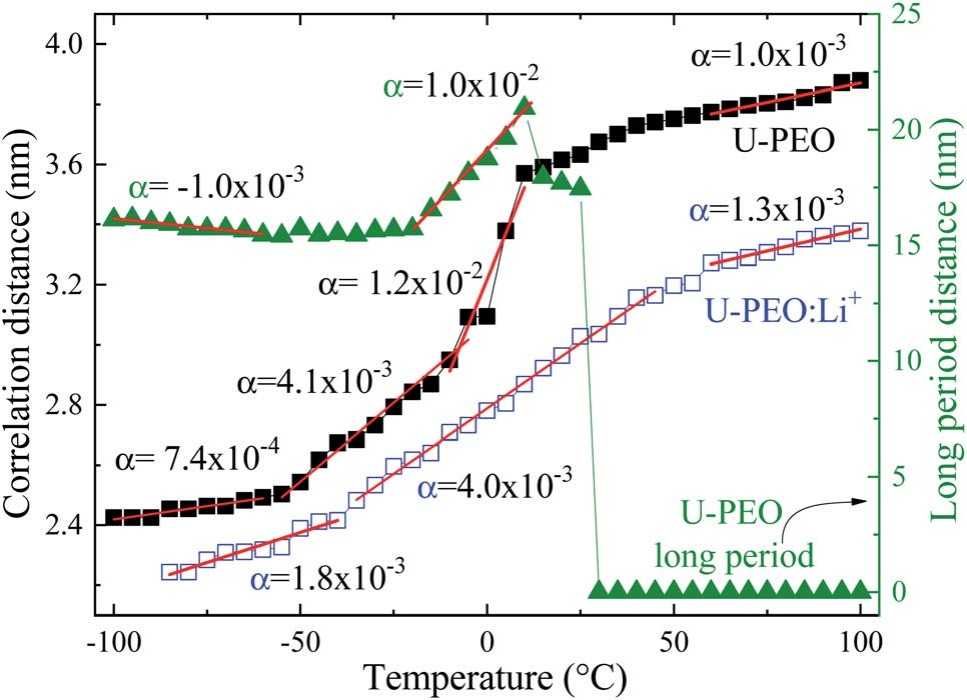
Siloxane correlation distance (*d*_sil_) for U-PEO and U-PEO:Li^+^, and the U-PEO long-period distance (*d*_sc_), as a function of temperature.

Fig. 7 shows the evolution, according to temperature, of the average correlation distance of the siloxane crosslink nodes (*d*_sil_) of U-PEO and U-PEO:Li^+^, calculated from the maximum peak position (*q*_max_) and the long-distance period of U-PEO (*d*_sc_). The very low thermal expansion (TE) of *d*_sil_ below *T*_g_ was consistent with the rigidity of the polymeric framework in the glass state, which relaxed and expanded at *T* > *T*_g_. The crossover of these TE regimes was in agreement with the *T*_g_ value found from the DSC analysis. A notable feature was the thermal shrinkage of *d*_sc_ below *T*_g_, which was consistent with the theoretical lattice dynamics prediction of negative thermal expansion along the oriented chain axis inside the lamellar crystalline phase.^56–59^ Above *T*_g_ and below 0 °C, the TE slopes for U-PEO and U-PEO:Li^+^ tended to approach the same final value, leading to a comparable value of the coefficient of linear thermal expansion (*α* = 4.0 × 10^−3^ °C^−1^). This finding indicated that the amorphous phase controlled the overall expansion of the semi-crystalline U-PEO hybrid.^60^ However, the accelerated thermal expansion observed in the U-PEO correlation distance, in the temperature range between −10 °C and 10 °C, evidenced an additive effect on the crystalline phase, inducing a higher expansion regime of the long-period distance. Accordingly, similar values of the coefficient of linear thermal expansion were obtained from the correlation distance (*α* = 1.2 × 10^−2^ °C^−1^) and the long-period distance (*α* = 1.0 × 10^−2^ °C^−1^). In contrast, the sharp decrease and complete disappearance of the long-period contribution for *T* > 25 °C coincided with the onset of the new linear regime of *d*_sil_, with *α* = 1.2 × 10^−3^ °C^−1^. These thermal events suggested that despite exclusion of the siloxane nodes from the PEO crystalline lamellae, the rapid expansion of the amorphous region between the lamellae permitted modulation of the mesh size and the free volume of the network, leading to greater segmental motion of the chains. Furthermore, for U-PEO:Li^+^, the higher temperature region displayed a linear TE regime with *α* value close to that observed for the U-PEO host matrix above the PEO melting temperature (*α* = 1.0 × 10^−3^ °C^−1^). This similarity indicates a decoupling of Li^+^-EO complexion of the segmental dynamics of PEO chains, inducing an additional contribution to the PEO conformational memory effect, associated with the *gauche* → *trans* conformation transition, which could also affect the Li^+^ mobility, as will be discussed in the next section.

### 3.3. Effect of temperature on the ionic conductivity of the U-PEO:Li^+^ SPE

In order to correlate the conformational and nanostructural characteristics with the macroscopic properties of U-PEO:Li^+^, AC impedance spectroscopy was used to study the evolution of the ionic conductivity as a function of temperature. Analysis at temperature lower than the melting point is important for better understanding of the global ionic conduction mechanism, because the natural multi-phases of semi-crystalline PEO can be a major drawback in this kind of SPE, where cation mobility is assisted by local relaxation and segmental motion of the amorphous region of the polymer chains.^61^

**Fig. 8:**
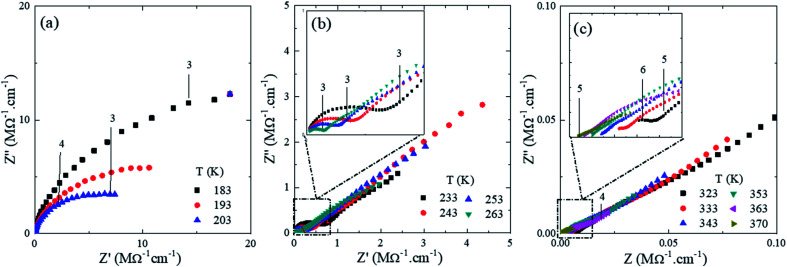
Nyquist impedance diagrams (*Z*′′ *vesus Z*′) for the U-PEO:Li^+^ OIH electrodes at different temperature (K) range (a) *T* ≤ 203, (b) 203 < *T* ≤ 263 and (c) *T* ≥ 323.

Fig. 8 shows the Nyquist impedance diagrams (*Z*′′ *versus Z*′) for U-PEO:Li^+^, normalized by the geometric factor, in the temperature range from 183 to 370 K (−90 to 100 °C). The numbers (between 3 and 6, shown in the insets of Fig. 8) represent the frequency range (from 1 kHz to 1 MHz), expressed in terms of log_10_ (signal frequency). The Nyquist diagrams at *T* < *T*_g_ (Fig. 8(a)) showed an incomplete semicircle, due to the high resistivity associated with the PEO polymer chain rigidity. For *T*_g_ < *T* < 15 °C (Fig. 8(b)), the semicircle for the U-PEO:Li^+^ SPE became complete and a linear dependency was observed in the low frequency region (3), characteristic of diffusion-controlled transport through the electrolyte, indicating increases of the free volume and the flexibility of the polymer chains as the temperature increased.^62^ On the other hand, for *T* > 15 °C, the SPE acted as an ionic liquid electrolyte (Fig. 8(c)).^63–65^ In this case, the semicircles were incomplete at the high frequency side (5), indicating that the spectral frequency used was not sufficiently high to follow the increase of the cation mobility. It has been reported previously that the high frequency semicircle does not appear in impedance plots for ion-facilitated plasticized polymer electrolytes.^44^ It is important to note that the temperature range in which the semicircle disappeared coincided with the melting point of the U-PEO, as observed in the thermal, vibrational, and nanostructural analyses. The exponential increases of the ionic conductivity observed up to this temperature range suggested the existence of the same conformational/nanostructural memory effect of the host U-PEO on the macroscopic ionic transport properties of U-PEO:Li^+^. Similar effects were observed elsewhere, with a sudden increase in ionic conductivity as the PEO *T*_m_ was approached, related to the PEO semi-crystalline (*gauche*) → amorphous (*trans*) phase transition.^43^

**Fig. 9:**
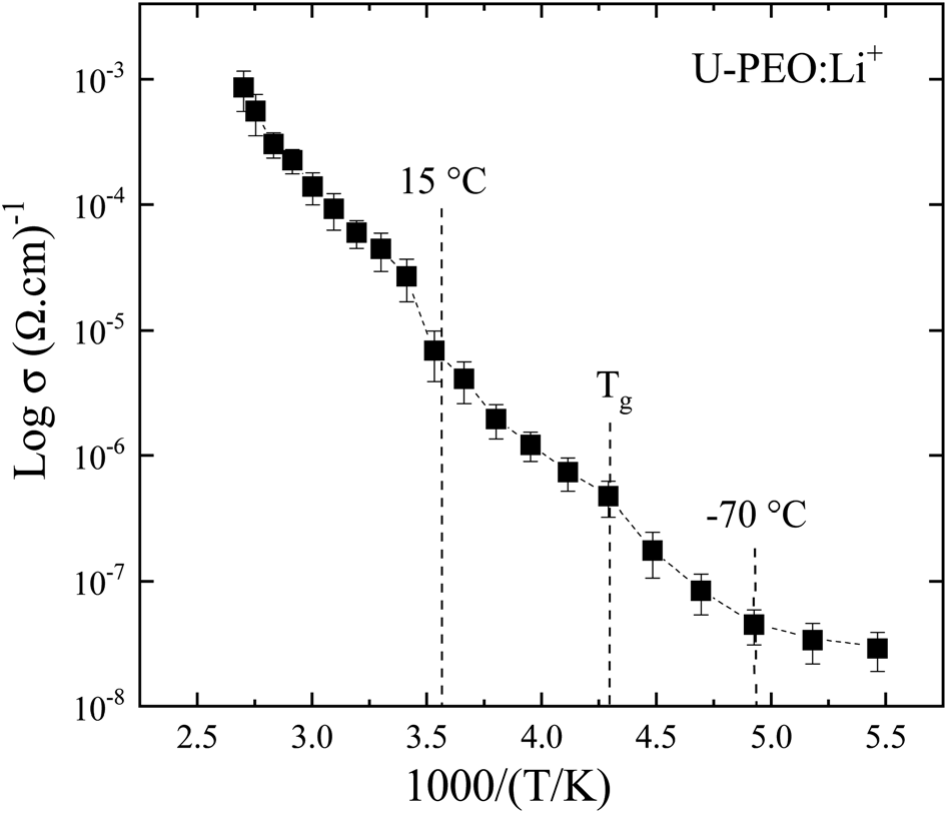
Arrhenius plot showing the conductivity dependence with reciprocal temperature of the U-PEO:Li^+^ SPE.

In order to correlate the thermo-structural transformation with the ionic transfer properties of U-PEO:Li^+^, the Arrhenius model was applied in the entire temperature range: σ = σ_∞_ exp−E^_a_^/kT, where *σ*_∞_ is a pre-exponential factor, *T* is the temperature, *E*_a_ is the activation energy of the conduction process, and *k* is the Boltzmann constant. As shown in Fig. 9, the U-PEO:Li^+^ SPE did not follow a single Arrhenius behavior in the entire range of temperature studied. A stepped pattern was also evident in the logarithmic dependency of conductivity with the temperature (Fig. SI.4†): (i) from −90 to −70 °C, the conductivity was almost invariant, which was consistent with the rigidity of the PEO polymer in the glass state (*T* < *T*_g_); (ii) for −60 °C < *T* < ∼15 °C, a linear regime was observed, with *E*_a_ = 0.35 eV, indicating a correlation between the ionic conduction and increase of the PEO free volume, due to the thermal expansion that occurred above −60 °C for both U-PEO and U-PEO:Li^+^ (Fig. 7). The onset of the ionic conductivity increased just above the glass transition of U-PEO (*T*_g_ = −58 °C) and below that of U-PEO:Li^+^ (*T*_g_ = −41 °C), as found from the DSC analyses, suggesting a memory effect of the segmental motion of the polymer chains of the U-PEO host on the ionic mobility of the U-PEO:Li^+^. The facilitated lithium motion could be explained by the segmental motion of the same chains with low numbers of ether oxygens coordinated to Li^+^ 3,57; (iii) for *T* > 20 °C, the linear ionic conductivity regime was more extended, with the highest slope (*E*_a_ = 0.45 eV).

This increase of the activation energy suggested a change of the ion transport mechanism, as a result of the Li^+^-EO bonds in the distinct U-PEO conformational backbone.^66^ In addition, the sharp conductivity change at the onset of this regime (∼15–20 °C) was consistent with decrease of the Li^+^-EO bond lifetime, due to the mobility of Li^+^ facilitated by the *trans* conformation of the polyether, leading to high mobility of Li^+^ cations, due to the segmental motion of the polymer chains. In the case of semi-crystalline SPE, this phenomenon is a consequence of the amorphous phase increasing at high temperature, with the polymer chain gaining faster internal modes in which bond rotation also contributes to the segmental motion.^67^ This then favors inter-chain and intra-chain ion movements, with the conductivity of the polymer electrolyte behaving in the same way as for a liquid-like electrolyte.^68,69^ The occurrence of these conductivity regime changes for U-PEO:Li^+^, at the same temperature as for the structural transformation caused by the melting (Fig. 7) of semi-crystalline U-PEO, was another indication of a memory effect associated with the motion of Li^+^ inside the cylindrical tunnels. Although conduction through the channels of the crystalline structure is well known, the notion of similar motion in isolated cylindrical channels in an amorphous matrix is a more recent concept.^70–72^ Accordingly, the accelerated increase of the *trans* conformation observed in this temperature range (Fig. 3(b)) was indicative of disorganization of the tunnels formed by lithium complexed by two interlocked chains. This phenomenon favors the decoupling of ionic conductivity from the segmental dynamics of the chains.^73^

## 4. Conclusions

An in-depth thermal, conformational, and nanostructural analysis was made of organic–inorganic hybrid monoliths based on ureasil–poly(ethylene oxide) (U-PEO, *M*_w_ = 1900 g mol^−1^), unloaded and loaded with Li^+^ (U-PEO:Li^+^, [EO]/[Li^+^] = 15). The Li^+^ cations were preferentially solvated by the ether-type oxygens of the polyether chains, in a crown-ether conformation. The intermolecular and intramolecular (Li-OEt) interactions increased the rigidity of the polymer chains, increasing the *T*_g_ value. The Li^+^-loaded SPE showed absence of the characteristic semi-crystalline U-PEO melting point (*T*_m_ = 29 °C).

The *T*_g_ and *T*_m_ of the host U-PEO matrix were marked by abrupt changes of the (O–C–C–O) backbone conformation of the PEO polymer chain. Below *T*_m_, the U-PEO matrix had a mainly *gauche* backbone conformation, clearly evidenced by the Raman bands located at 280 and 360 cm^−1^, which disappeared near *T*_m_. The U-PEO:Li^+^ spectrum showed comparable amounts of *trans* and *gauche* (O–C–C–O) backbone below *T*_g_, evidencing inter-chain interactions arising from solvation of the lithium cations. An accelerated increase of the *trans* (O–C–C–O) component was observed above the U-PEO melting point, indicating disorganization of the tunnels formed by complexation of the lithium atoms with the interlocked chain segments, induced by a thermal decomplexation effect. The comparable numbers of acceleration events (similar to ‘bottlenecks’) observed in the thermal evolution of the *trans* and *gauche* bands evidenced a memory effect of the Li^+^-free polymeric chains in the (O–C–C–O) backbone conformation of the U-PEO:Li^+^ SPE.

The linear thermal expansion of the average correlation distance between the siloxane nodes and the long-distance period of the lamellar semi-crystalline edifice were determined by SAXS. Comparison of the expansion curves indicated that the siloxane nodes were excluded from the PEO crystalline edifice of the semi-crystalline U-PEO, while the sharp expansion of the amorphous region between the lamellae, during melting, enabled modulation of the hybrid network free volume. Far from the *T*_m_, comparable thermal expansion coefficients were found for U-PEO and U-PEO:Li^+^, indicating a dominant contribution of the amorphous phase in the semi-crystalline sample.

Another indication of the effect of semi-crystalline structure memory on the U-PEO:Li^+^ SPE properties was revealed by the temperature-dependence of the ionic conductivity. For the amorphous sample, three different regimes were observed in the Arrhenius plot of conductivity, with a pronounced deviation of the linear behavior above *T*_m_, evidencing a role of temperature-induced Li^+^-EO decomplexation in the ionic conduction process.

The set of results related to the key role of Li^+^-EO bonds in the distinct U-PEO conformational backbone may inspire the materials chemistry scientists to design optimized structures of polymer electrolytes with minimized structural memory of crystalline building blocks and improved ionic conductivity.

## Author contributions

The manuscript was written through contributions of all authors. All authors have given approval to the final version of the manuscript.

## Conflicts of interest

There are no conflicts to declare.

## Supplementary Material

RA-012-D1RA09138G-s001

## References

[cit1] Zhang H., Li C., Piszcz M., Coya E., Rojo T., Rodriguez-Martinez L. M., Armand M., Zhou Z. (2017). Chem. Soc. Rev..

[cit2] Xue Z., He D., Xie X. (2015). J. Mater. Chem. A.

[cit3] Palácio G., Pulcinelli S. H., Mahiou R., Boyer D., Chadeyron G., Santilli C. V. (2018). ACS Appl. Mater. Interfaces.

[cit4] Quartarone E., Mustarelli P., Magistris A. (1998). Solid State Ionics.

[cit5] Kim J., Park D.-W., Soo-Jin P., Kim S. (2013). Res. Chem. Intermed..

[cit6] Phan T. N. T., Issa S., Gigmes D. (2019). Polym. Int..

[cit7] Godovsky Y. K., Slonimsky G. L., Garbar N. M. (1972). J. Polym. Sci., Part C: Polym. Symp..

[cit8] Jiang Y., Yan X., Ma Z., Mei P., Xiao W., You Q., Zhang Y. (2018). Polymers.

[cit9] Wang Q., Zhang H., Cui Z., Zhou Q., Shangguan X., Tian S., Zhou X., Cui G. (2019). Energy Storage Materials.

[cit10] Zhao Y., Tao R., Fujinami T. (2006). Electrochim. Acta.

[cit11] Liu T. M., Saikia D., Ho S. Y., Chen M. C., Kao H. M. (2017). RSC Adv..

[cit12] Chaker J. A., Santilli C. V., Pulcinelli S. H., Dahmouche K., Briois V., Judeinstein P. (2007). J. Mater. Chem..

[cit13] Gray F. M. , Solid Polymer Electrolytes: Fundamentals and Technological Applications, VHC Publishers, New York, 1st edn, 1991

[cit14] Hall P. G., Davies G. R., Ward I. M., McIntyre J. E. (1986). Polym. Commun..

[cit15] Fish D., Khan I., Smid J. (1986). Die Makromolekulare Chemie Rapid Communications.

[cit16] Spindler R., Shriver D. F. (1988). J. Am. Chem. Soc..

[cit17] Rodrigues D. E., Brennan A. B., Betrabet C., Wanga B., Wilkes G. L. (1992). Chem. Mater..

[cit18] Saikia D., Pan Y. C., Wu C. G., Fang J., Tsai L. D., Kao H. M. (2014). J. Mater. Chem. C.

[cit19] Zhang H., Li C., Piszcz M., Coya E., Rojo T., Rodriguez-Martinez L. M., Armand M., Zhou Z. (2017). Chem. Soc. Rev..

[cit20] Ghosh A., Wang C., Kofinas P. (2010). J. Electrochem. Soc..

[cit21] Li X., Cheng S., Zheng Y., Li C. Y. (2019). Mol. Syst. Des. Eng..

[cit22] Dahmouche K., de Souza P. H., Bonagamba T. J., Paneppucci H., Judeinstein P., Pulcinelli S. H., Santilli C. V. (1998). J. Sol-Gel Sci. Technol..

[cit23] Dahmouche K., Santilli C. V., da Silva M., Ribeiro C. A., Pulcinelli S. H., Craievich A. F. (1999). J. Non-Cryst. Solids.

[cit24] Hashimoto A., Inoue J., Funatomi T., Minoh M. (2016). Int. J. Hum.-Comput. Interact..

[cit25] Labrèche C., Lévesque I., Prud'homme J. (1996). Macromolecules.

[cit26] Edman L., Ferry A., Doeff M. M. (2000). J. Mater. Res..

[cit27] Reiter G. (2020). J. Chem. Phys..

[cit28] Chandran S., Reiter G. (2019). ACS Macro Lett..

[cit29] Shakhnovich E. I., Gutin A. M. (1989). J. Phys. A: Math. Gen..

[cit30] Tanaka T., Wang C., Pande V., Grosberg A. Y., English A., Masamune S., Gold H., Levy R., King K. (1995). Faraday Discuss..

[cit31] Bryngelson J. D., Wolynes P. G. (1987). Proc. Natl. Acad. Sci. U. S. A..

[cit32] Shakhnovich E. I., Gutin A. M. (1993). Proc. Natl. Acad. Sci. U. S. A..

[cit33] Wolynes P. G., Onuchic J. N., Thirumalai D. (1995). Science.

[cit34] Pande V. S., Grosberg A. Y., Tanaka T. (1994). Proc. Natl. Acad. Sci. U. S. A..

[cit35] Alvarez-Lorenzo C., Guney O., Oya T., Sakai Y., Kobayashi M., Enoki T., Takeoka Y., Ishibashi T., Kuroda K., Tanaka K., Wang G., Grosberg A. Y., Masamune S., Tanaka T. (2000). Macromolecules.

[cit36] Wang Y., Fan F., Agapov A. L., Saito T., Yang J., Yu X., Hong K., Mays J., Sokolov A. P. (2014). Polymer.

[cit37] Gao J., Wang C., Han D.-W., Shin D.-M. (2021). Chem. Sci..

[cit38] Santilli C. V., Chiavacci L. A., Lopes L., Pulcinelli S. H., Oliveira A. G., Cie F. (2009). Chem. Mater..

[cit39] Dahmouche K., Carlos L. D., Santilli C. V., Bermudez D. Z. (2002). J. Phys. Chem. B.

[cit40] Trentin A., Harb S. V., Uvida M. C., Marcoen K., Pulcinelli S. H., Santilli C. V., Terryn H., Hauffman T., Hammer P. (2021). Corros. Sci..

[cit41] Strawhecker K. E., Manias E. (2003). Chem. Mater..

[cit42] Vaia R. A., Teukolsky R. K., Giannelis E. P. (1994). Chem. Mater..

[cit43] Chaurasia S. K., Chandra A. (2017). Solid State Ionics.

[cit44] Herstedt M., Smirnov M., Johansson P., Chami M., Grondin J., Servant L. (2005). J. Raman Spectrosc..

[cit45] Rey I., Johansson P., Lindgren J., Lassègues J. C., Grondin J., Servant L. (1998). J. Phys. Chem. A.

[cit46] Yoshihara T., Tadokoro H., Murahashi S. (1964). J. Chem. Phys..

[cit47] Miyazawa T., Fukushima K., Ideguchi Y. (1962). J. Chem. Phys..

[cit48] Maxfield J., Shepherd I. W. (1975). Polymer.

[cit49] Rieppo L., Saarakkala S., Närhi T., Helminen H. J., Jurvelin J. S., Rieppo J. (2012). Osteoarthr. Cartil..

[cit50] Papke B. L., Ratner M. A., Shriver D. F. (1981). J. Phys. Chem. Solids.

[cit51] Dahmouche K., Santilli C. V., Pulcinelli S. H., Craievich A. F. (1999). J. Phys. Chem. B.

[cit52] Schnablegger H. and Singh Y., The SAXS Guide: Getting acquainted with the principles, Anton Paar GmbH, Austria, 3rd edn, 2013

[cit53] Papke B. L., Ratner M. A. (1981). J. Phys. Chem. Solids.

[cit54] Suzuki Y., Duran H., Steinhart M., Butt H., Floudas G. (2014). Macromolecules.

[cit55] Tonami K., Nojima S., Honda T., Tsunogae Y. (2009). Polym. J..

[cit56] White G. K., Choy C. L. (1984). J. Polym. Sci., Polym. Chem. Ed..

[cit57] Kurita T., Fukuda Y., Takahashi M., Sasanuma Y. (2018). ACS Omega.

[cit58] Choy C. L., Wong S. P., Young K. (1984). J. Polym. Sci., Polym. Chem. Ed..

[cit59] Chen F. C., Choy C. L., Young K. (1980). J. Polym. Sci., Polym. Chem. Ed..

[cit60] Known K., Isayev A. I., Kim K. H., van Sweden C. (2006). Polym. Eng. Sci..

[cit61] Choi J., Cheruvally G., Kim Y., Kim J., Manuel J., Raghavan P., Ahn J., Kim K., Ahn H. (2007). Solid State Ionics.

[cit62] Jung Y., Park M., Doh C., Kim D. (2016). Electrochim. Acta.

[cit63] Reddy C. V. S., Sharma A. K., Rao V. V. R. N. (2003). J. Power Sources.

[cit64] Xi J., Qiu X., Ma X., Cui M., Yang J., Tang X., Zhu W., Chen L. (2005). Solid State Ionics.

[cit65] Michael M. S., Jacob M. M. E., Prabaharan S. R. S., Radhakrishna S. (1997). Solid State Ionics.

[cit66] Aziz S. B., Woo T. J., Kadir M. F. Z., Ahmed H. M. (2018). J. Sci.: Adv.
Mater. Devices.

[cit67] Fullerton-shirey S. K., Maranas J. K. (2009). Macromolecules.

[cit68] Palacio G., Pulcinelli S. H., Mahiou R., Boyer D., Chadeyron G., Santilli C. V. (2018). ACS Appl. Mater. Interfaces.

[cit69] Sreekanth T., Reddy M. J., Ramalingaiah S., Rao U. V. S. (1999). J. Power Sources.

[cit70] Salas-De La Cruz D., Green M. D., Ye Y., Elabd Y. A., Long T. E., Winey K. I. (2012). J. Polym. Sci., Part B: Polym. Phys..

[cit71] Ramesh S., Liew C. W., Ramesh K. (2011). J. Non-Cryst. Solids.

[cit72] Wohlmuth D., Epp V., Bottke P., Hanzu I., Bitschnau B., Letofsky-Papst I., Kriechbaum M., Amenitsch H., Hofer F., Wilkening M. (2014). J. Mater. Chem. A.

[cit73] Money B. K., Hariharan K., Swenson J. (2014). Solid State Ionics.

