# Supplementary material for: Fingerprint of semi-crystalline structure memory in the thermal and ionic conduction properties of amorphous ureasil–polyether hybrid solid electrolytes
Source: RSC Adv. 2022 Feb 14;12(9):5225–35. doi: 10.1039/d1ra09138g (PMC8981479; doi:10.1039/d1ra09138g)
Supplement: RA-012-D1RA09138G-s001 [file RA-012-D1RA09138G-s001.pdf]

Electronic Supplementary Material (ESI) for RSC Advances.
This journal is © The Royal Society of Chemistry 2022

Supporting Information

Fingerprint of semi-crystalline structure memory in the thermal and ionic conduction properties of amorphous ureasil-polyether hybrid solid electrolytes

Gustavo Palácio †, Sandra H. Pulcinelli †, Celso V. Santilli †*

† Chemistry Institute of the São Paulo State University – UNESP, 14800-060 Araraquara, São Paulo, Brazil.

Corresponding author. Phone: +55-016-3301-9546. fax: +55-016-3322-2308

E-mail: cv.santilli@unesp.br

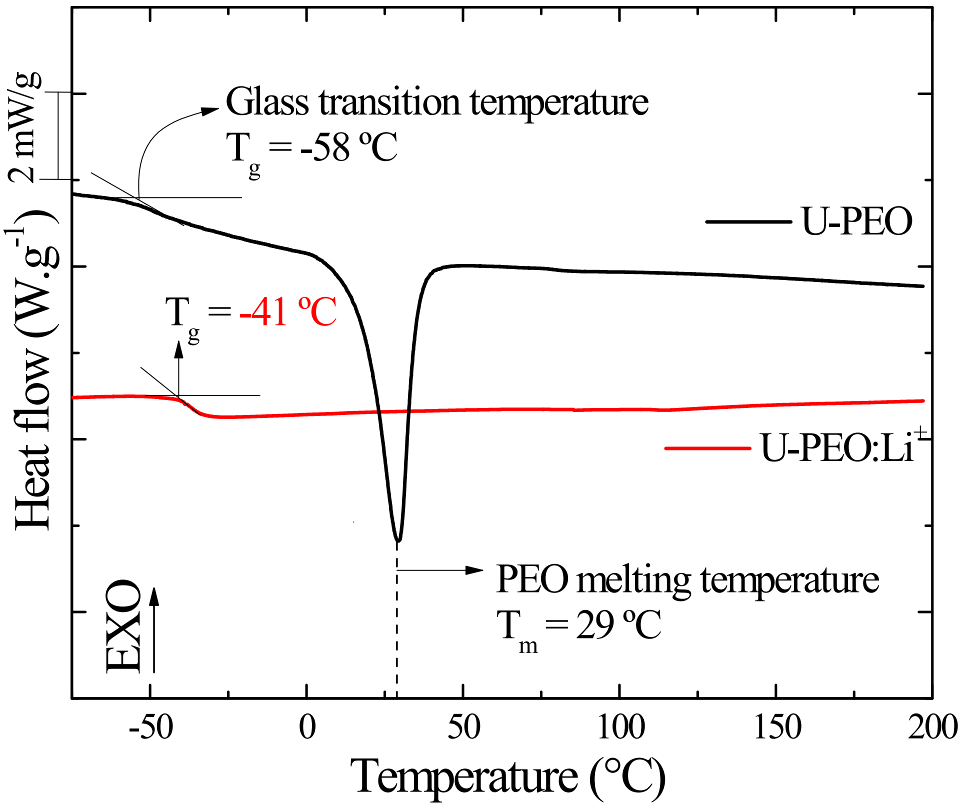

Figure SI.1: DSC curves of U-PEO and of U-PEO:Li^+ samples.

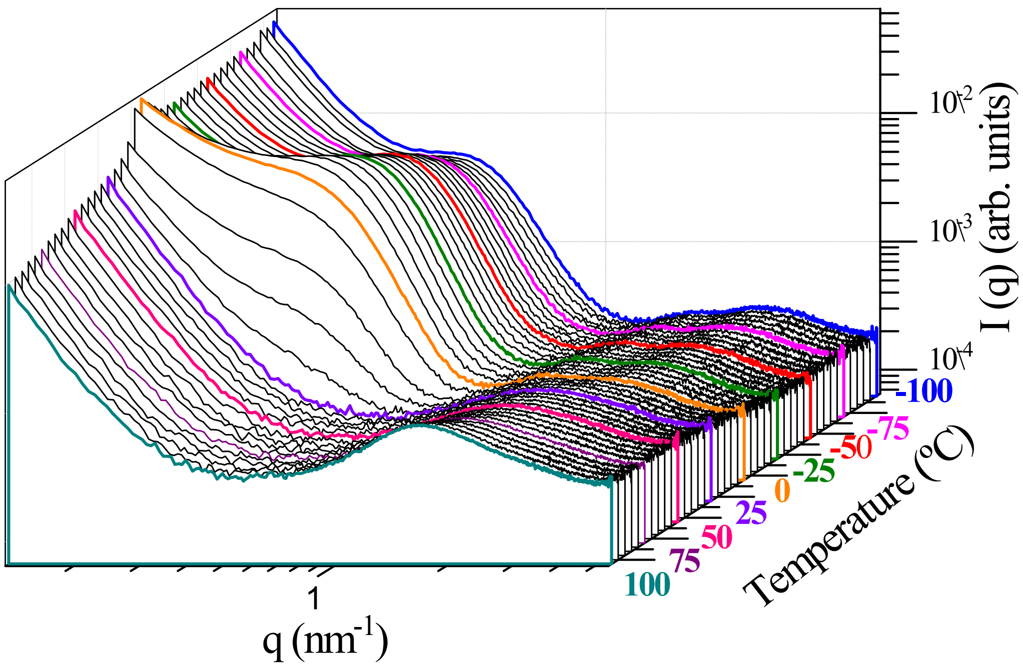

Figure SI.2: Stacked log-log SAXS curves for U-PEO, as a function of temperature, recorded in situ during heating from 100 ºC to -100 ºC.

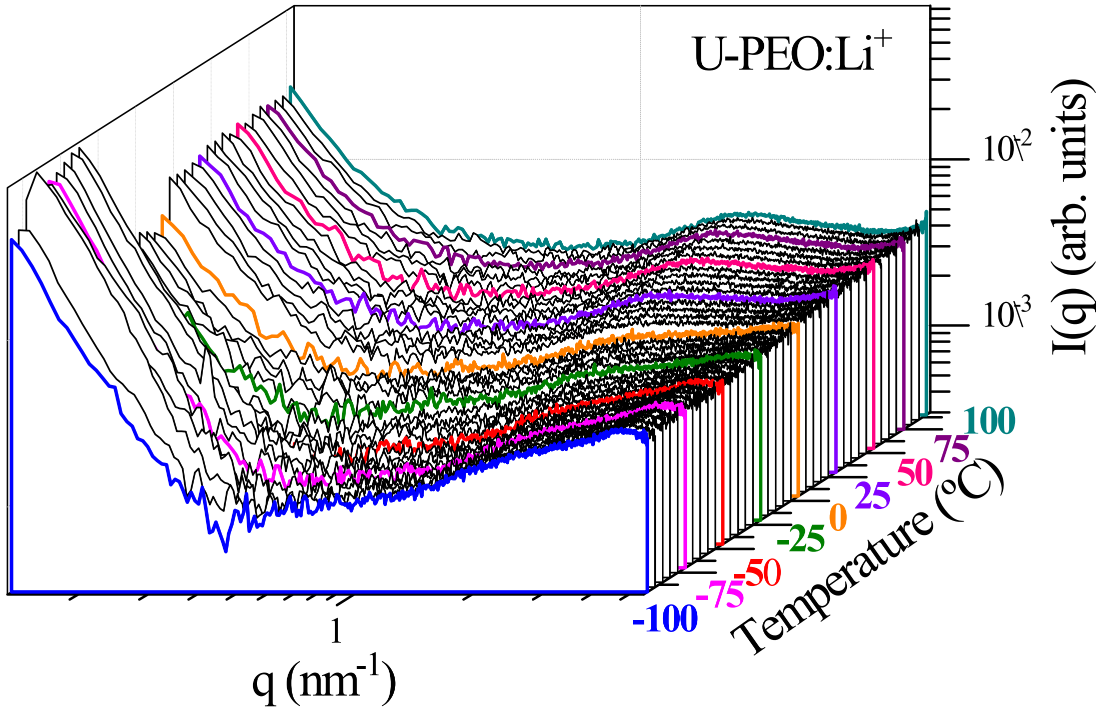

Figure SI.3: Stacked log-log SAXS curves for U-PEO:Li^+, as a function of temperature, recorded *in situ* during heating from -100 ºC to 100 ºC.

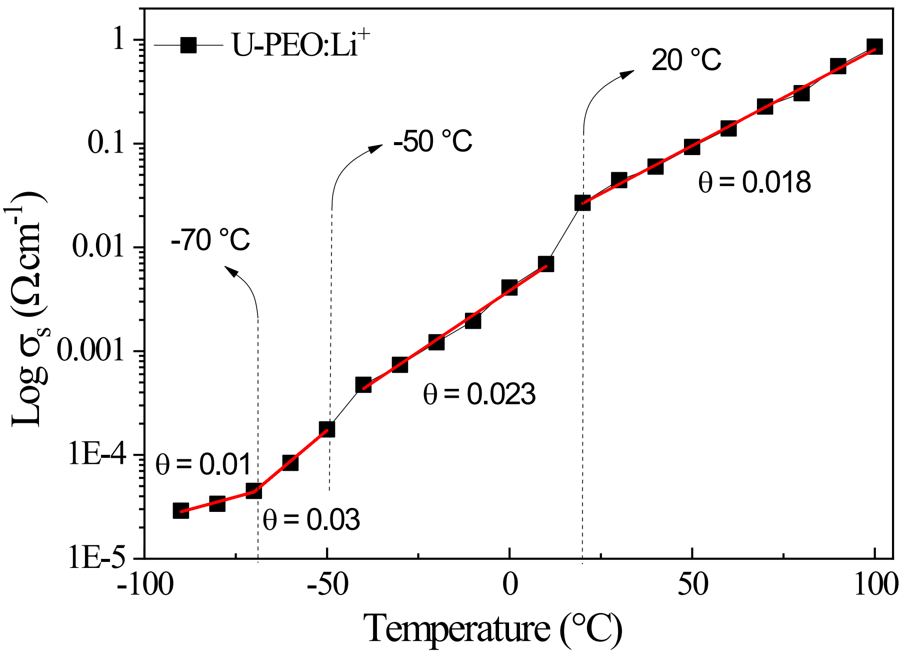

Figure SI.4: Logarithmic dependency of conductivity with temperature of the U-PEO:Li^+.
